# Analysis of clinical efficacy of remifentanil combined with propofol anesthesia in patients with traumatic brain injury

**DOI:** 10.3389/fneur.2026.1868176

**Published:** 2026-07-14

**Authors:** Zhenyu Fan, Ling Wang, Wenjuan Wang, Hui Yang, Nina Chen, Ting Gong

**Affiliations:** 1 Department of Anesthesiology, Xiangyang No.1 People's Hospital, Hubei University of Medicine, Xiangyang, China; 2 Department of Nursing, Xiangyang No.1 People's Hospital, Hubei University of Medicine, Xiangyang, China; 3 Department of Anesthesia Care, Xiangyang No.1 People's Hospital, Hubei University of Medicine, Xiangyang, China

**Keywords:** clinical effect, combined anesthesia, propofol, remifentanil, traumatic brain injury

## Abstract

**Objective:**

This study aims to analyze the clinical effect of remifentanil combined with propofol in anesthesia for patients with traumatic brain injury.

**Methods:**

The study design is a retrospective analysis, incorporating the medical records of 110 patients with traumatic brain injury treated in our hospital from January 2024 to December 2024. According to different anesthesia regimens, the patients were divided into a remifentanil combined with propofol group (study group, *n* = 55) and a propofol group (control group, *n* = 55). The vital signs, anesthesia efficacy, Visual Analog Scale (VAS) scores, Riker Sedation-Agitation Scale (RSAS) scores, Glasgow Coma Scale (GCS) scores, and stress response levels of the two groups were monitored to comprehensively analyze the clinical effects.

**Results:**

The results showed no significant differences in the general data between the two groups, indicating comparability (*P* > 0.05). There were no statistically significant differences in post-medication body temperature, heart rate (HR), mean arterial pressure (MAP), and peripheral oxygen saturation (SpO_2_) levels between the two groups (*P* > 0.05). The incidence rates of agitation, stress response, and respiratory and circulatory depression in the study group were all lower than those in the control group, while the successful intubation rate was higher (*P* < 0.05). The VAS and RSAS scores of the study group at 12 h and 24 h post-medication were significantly lower than those of the control group (*P* < 0.05). The norepinephrine (NE) and angiotensin II (Ang II) levels at extubation in the study group were lower than those in the control group (*P* < 0.05). During follow-up, there was no statistically significant difference in GCS scores between the two groups (*P* > 0.05).

**Conclusion:**

The study confirms that remifentanil combined with propofol has significant clinical effects in anesthesia for patients with traumatic brain injury. It can effectively alleviate perioperative pain and agitation in patients with traumatic brain injury and reduce stress responses, making it worthy of clinical promotion.

## 1 Introduction

Traumatic Brain Injury (TBI) remains a leading cause of death and long-term disability worldwide. Its pathophysiology involves primary mechanical damage and secondary cascades including cerebral ischemia, neuroinflammation, and oxidative stress (1–3). Patients with TBI are at risk of life-threatening complications such as elevated intracranial pressure, cerebral hypoperfusion, and systemic inflammatory response syndrome (SIRS), all of which impose stringent demands on anesthetic management (4, 5). Conventional anesthesia protocols must balance neuroprotection, hemodynamic stability, and rapid postoperative emergence. However, single-agent regimens often fail to fully address these clinical requirements.

Remifentanil, an ultra-short-acting μ-opioid receptor agonist, offers advantages including rapid onset and organ-independent metabolism, though its sole use may carry risks of hyperalgesia and respiratory depression. Propofol, an intravenous anesthetic, exerts sedative-hypnotic effects through γ-aminobutyric acid (GABA) receptor modulation while providing anticonvulsant and antioxidant properties, though its use requires careful titration because of the risk of dose-dependent hypotension in TBI patients.

In recent years, multimodal drug strategies have emerged as a focus in optimizing TBI anesthesia (6, 7). The combination of remifentanil and propofol may offer synergistic benefits through complementary mechanisms. Remifentanil attenuates stress responses by suppressing sympathetic activity, whereas propofol may reduce secondary brain injury through cerebrovascular regulation (8–10). However, existing evidence primarily comes from studies in healthy volunteers or non-TBI surgical populations, with limited systematic evaluation of this combination specifically in TBI patients.

This retrospective study analyzed medical records of 110 TBI patients admitted from January 2024 to December 2024, comparing anesthetic outcomes between remifentanil-propofol combination therapy (study group) and propofol monotherapy (control group). The primary objectives were to evaluate the impact of combination therapy on perioperative pain control, stress responses, and neurological outcomes. We hypothesized that the synergistic effects of the two agents would reduce agitation incidence, attenuate stress reactivity, and maintain hemodynamic stability in TBI patients, thereby providing evidence to guide clinical anesthesia practice.

## 2 Materials and methods

### 2.1 Study design

This single-center retrospective cohort study included TBI patients who underwent neurosurgical intervention at our institution between January 2024 and December 2024. After applying the inclusion and exclusion criteria, 110 patients were enrolled and divided into either the study group (remifentanil + propofol, *n* = 55) or the control group (propofol alone, *n* = 55). The study adhered to the ethical principles of the Declaration of Helsinki and complied with national medical data protection regulations. The study protocol was reviewed and approved by the Institutional Review Board of Xiangyang No.1 People's Hospital (Approval No. 2023071). Patient confidentiality was maintained by de-identifying all records prior to data extraction, implementing tiered access controls to restrict full dataset access to authorized researchers, and presenting only aggregated clinical data in publications to eliminate individual identification risks. The Institutional Review Board waived the requirement for individual informed consent due to the retrospective nature of the study and the use of de-identified data.

### 2.2 Inclusion criteria

Patients were eligible if they: (1) received a definitive TBI diagnosis via cranial CT imaging combined with clinical symptoms and signs (11, 12); (2) were aged 18–65 years; (3) met anesthetic and surgical indications; (4) had complete and retrievable medical records.

Patients were excluded if they: (1) presented with severe cardiopulmonary dysfunction or other major systemic diseases; (2) had undergone preoperative tracheotomy; (3) were pregnant or lactating; (4) had organ failure (e.g., hepatic, renal, cardiac) or systemic metabolic disorders (e.g., diabetic ketoacidosis, thyroid storm); (5) had psychiatric disorders or cognitive impairments precluding clinical cooperation.

### 2.3 Anesthesia protocol

#### 2.3.1 Preoperative Preparation

All patients received standardized preoperative care, which included ensuring airway patency, establishing peripheral venous access, and instituting continuous monitoring of vital signs including heart rate, blood pressure, and peripheral oxygen saturation (SpO_2_). Targeted interventions were implemented according to individual needs, such as managing intracranial pressure or addressing potential cerebral herniation risks.

#### 2.3.2 Anesthesia Induction

General anesthesia was induced with weight-based intravenous dosing. Patients received propofol (Guangdong Jiabo Pharmaceutical, approval no. H20143355) at 2 mg/kg, fentanyl (Yichang Renfu Pharmaceutical, approval no. H20205068) at 6 μg/kg, and an adequate dose of rocuronium (Hainan Steda Pharmaceutical, approval no. H20203679) to achieve neuromuscular blockade. Following loss of consciousness, orotracheal intubation was performed and mechanical ventilation was initiated.

#### 2.3.3 Anesthesia Maintenance

For anesthesia maintenance, the control group received a continuous infusion of propofol at 4–8 mg/kg/h. In contrast, the study group received a combined infusion of propofol at the same rate alongside remifentanil (Jiangsu Nhwa Pharmaceutical, approval no. H20143315) at 0.25 μg/kg/min.

#### 2.3.4 Intraoperative Management

During surgery, the infusion rates were dynamically adjusted according to real-time vital signs. Anesthesia depth was further maintained with inhaled sevoflurane (Lunan Better Pharmaceutical, approval no. H20080681) at an end-tidal concentration of 1.5%−2.0%, and intermittent boluses of rocuronium were administered as needed for continued neuromuscular blockade. Sevoflurane was discontinued 20 min prior to surgical completion, with gradual tapering of the propofol infusion.

#### 2.3.5 Postoperative Recovery

After surgery, extubation was performed once spontaneous ventilation was deemed adequate, defined by the presence of regular respiratory rhythm and sufficient tidal volume. Patients were then transferred to the intensive care unit for continued postoperative monitoring.

### 2.4 Data collection

Baseline patient data [age, sex, Glasgow Coma Scale (GCS) score, injury type], intraoperative monitoring parameters [body temperature, heart rate (HR), mean arterial pressure (MAP), oxygen saturation (SpO_2_)], anesthesia efficacy indicators, pain scores, sedation scores, stress response biomarkers [post-extubation norepinephrine (NE) and angiotensin II (Ang II) levels], and postoperative follow-up GCS scores were collected.

Anesthesia efficacy was evaluated using a standardized clinical indicator system encompassing four core parameters: incidence of postoperative agitation, frequency of stress responses, rate of respiratory and circulatory depression, and success rate of tracheal intubation. Pain intensity was quantified using the Visual Analog Scale (VAS) at three critical time points (1, 12, and 24 h post-drug administration). The VAS employs a 0–10 continuous scoring system, with scores directly proportional to subjective pain severity.

Behavioral agitation was assessed via the Richmond Agitation-Sedation Scale (RASS) at identical postoperative intervals. The RASS is a 10-level scale ranging from +4 (combative) to−5 (unarousable), with 0 representing an alert and calm state. Neurological outcome prognosis was determined using the Glasgow Outcome Scale (GOS), a 5-tiered classification: 1 = death; 2 = vegetative state; 3 = severe disability; 4 = moderate disability; 5 = full recovery with independent functioning.

### 2.5 Statistical analysis

Statistical analyses were performed using SPSS version 26.0. Continuous variables are presented as mean ± standard deviation (x±s) and compared between groups using independent samples *t*-tests. Categorical variables are expressed as frequencies (percentages) and analyzed using chi-square (χ^2^) tests. A *p*-value < 0.05 was considered statistically significant.

## 3 Results

### 3.1 Baseline characteristics

A total of 110 patients were included in the final analysis, with 55 patients in each group. The control group consisted of 31 males and 24 females, with a mean age of 40.02 ± 2.94 years (range: 25–60) and a mean BMI of 20.11 ± 0.58 kg/m^2^ (range: 18–21). The study group comprised 33 males and 22 females, with a mean age of 39.91 ± 2.73 years (range: 25–60) and a mean BMI of 20.25 ± 0.43 kg/m^2^ (range: 18–21). Baseline characteristics were well balanced between the two groups, with no statistically significant differences observed (all *P* > 0.05) (Table 1).

**Table 1:** Baseline characteristics comparison between the two groups.

Characteristic	Category	Control group	Study group	*t/*χ2	*P*
*n*		55	55	–	–
Gender				0.150	0.699
	Male	31	33		
	Female	24	22		
Age	–	25–60	25–60	–	–
(years)	Mean	40.02 ± 2.94	39.91 ± 2.73	0.203	0.839
BMI	–	18–21	18–21	–	–
(kg/m^2^)	Mean	20.11 ± 0.58	20.25 ± 0.43	1.438	0.153

### 3.2 Intraoperative vital signs

No significant differences were detected between the two groups in postoperative body temperature, heart rate (HR), mean arterial pressure (MAP), or oxygen saturation (SpO_2_) (all *P* > 0.05) (Table 2).

**Table 2:** Intraoperative vital signs comparison between the two groups.

Vital sign parameter	Control group	Study group	*t*	*P*
*n*	55	55	–	–
Body temperature (°C)	36.28 ± 0.74	36.15 ± 0.54	1.052	0.295
HR (times/min)	77.05 ± 3.14	76.45 ± 3.53	0.942	0.348
MAP(mmHg)	77.96 ± 3.78	78.28 ± 3.66	0.451	0.653
SpO2 (%)	98.05 ± 1.25	98.14 ± 1.14	0.395	0.694

### 3.3 Anesthesia efficacy

The remifentanil-propofol combination was associated with superior anesthesia outcomes compared to propofol monotherapy. Specifically, the study group exhibited significantly lower rates of agitation (3.64% vs. 16.36%), stress responses (1.82% vs. 14.55%), and respiratory/circulatory depression (0.00% vs. 10.91%), as well as a higher successful intubation rate (96.36% vs. 81.82%) (all *P* < 0.05) (Table 3).

**Table 3:** Anesthesia efficacy comparison between the two groups.

Control group	Study group	χ2	*P*	*P* value
*n*	55	55	–	–
Agitation				
*n*	9	2	4.95	0.026
Rate (%)	16.36	3.64		
Stress response				
*n*	8	1	4.356	0.037
Rate (%)	14.55	1.82		
Respiratory and circulatory depression				
*n*	6	0	4.407	0.036
Rate (%)	10.91	0		
Successful intubation				
*n*	45	53	5.986	0.014
Rate (%)	81.82	96.36		

### 3.4 Postoperative pain assessment

VAS scores were comparable between the two groups at 1h post-medication (*P* = 0.097). However, at 12 h and 24 h post-medication, the study group showed significantly lower VAS scores than the control group (12h: 2.88 ± 0.53 vs. 4.96 ± 0.88, *P* < 0.001; 24h: 4.13 ± 0.74 vs. 7.15 ± 1.14, *P* < 0.001) (Table 4).

**Table 4:** VAS scores at different time points post-drug administration.

Time point	Control group	Study group	*t*	*P*
*n*	55	55	–	–
1h after medication	2.25 ± 0.25	2.16 ± 0.31	1.676	0.097
12h after medication	4.96 ± 0.88	2.88 ± 0.53	15.016	< 0.001
24h after medication	7.15 ± 1.14	4.13 ± 0.74	16.479	< 0.001

### 3.5 Postoperative agitation assessment

RASS scores did not differ significantly at 1 h post-medication between the two groups (*P* = 0.091). In contrast, at 12 h and 24 h post-medication, the study group demonstrated markedly lower RASS scores compared to the control group (12h: 2.88 ± 0.21 vs. 3.73 ± 0.56, *P* < 0.001; 24h: 4.33 ± 0.85 vs. 5.01 ± 0.94, *P* < 0.001) (Table 5).

**Table 5:** RASS scores at different time points post-drug administration.

Time point	Control group	Study group	*t*	*P*
*n*	55	55	–	–
1h after medication	2.45 ± 0.14	2.39 ± 0.22	1.706	0.091
12h after medication	3.73 ± 0.56	2.88 ± 0.21	10.540	< 0.001
24h after medication	5.01 ± 0.94	4.33 ± 0.85	3.979	< 0.001

### 3.6 Stress response biomarkers

Post-extubation plasma levels of norepinephrine (NE) and angiotensin II (Ang II) were significantly lower in the study group than in the control group (NE: 361.23 ± 12.45 vs. 385.14 ± 15.41 pg/ml, *P* < 0.001; Ang II: 34.56 ± 2.11 vs. 40.41 ± 3.71 ng/L, *P* < 0.001) (Table 6). Reference ranges for healthy resting adults are 149–564 pg/ml for NE and ≤ 52 ng/L for Ang II, indicating that both groups exhibited elevated levels consistent with the hyperadrenergic state commonly observed in TBI patients.

**Table 6:** NE and Ang II levels at extubation between the two groups.

Control group	Study group	*t*	*P*	*P* value
*n*	55	55	–	–
NE (pg/ml)	385.14 ± 15.41	361.23 ± 12.45	8.951	< 0.001
Ang II (ng/L)	40.41 ± 3.71	34.56 ± 2.11	10.165	< 0.001

### 3.7 Neurological outcome

At the 3-month follow-up, GCS scores showed no statistically significant difference between the study group and the control group (4.45 ± 0.54 vs. 4.36 ± 0.13, *P* = 0.064) (Table 7).

**Table 7:** GCS scores at 3-month follow-up between the two groups.

Control group	Study group	*t*	*P*	*P* value
*n*	55	55	–	–
GCS	4.36 ± 0.13	4.45 ± 0.54	1.874	0.064

## 4 Discussion

Traumatic Brain Injury (TBI) results from external mechanical forces and remains a leading cause of mortality and long-term disability worldwide. The pathophysiological cascade of TBI includes primary mechanical damage and secondary insults such as cerebral ischemia, neuroinflammation, and oxidative stress (13, 14). During neurosurgical interventions, effective anesthetic management is critical for maintaining hemodynamic stability and preventing secondary brain injury (15–17). Remifentanil, an ultra-short-acting μ-opioid agonist, provides rapid analgesia with organ-independent metabolism, whereas propofol exerts sedative-hypnotic effects through GABAergic modulation and possesses neuroprotective properties. Preclinical and clinical evidence suggests that their combination may offer synergistic benefits by enhancing analgesia and sedation while reducing dose-related adverse effects (18, 19), a premise supported by the present findings.

Several mechanisms may explain the observed superiority of the remifentanil-propofol regimen over propofol alone. Remifentanil reduces sympathetic outflow and catecholamine release through central μ-receptor activation, thereby attenuating perioperative stress responses (10, 20). Propofol, in turn, decreases cerebral blood flow and intracranial pressure via cerebrovascular dilation, contributing to optimal surgical conditions. The concurrent administration of these agents allows for dose reduction of each drug, potentially minimizing propofol-related hypotension and remifentanil-induced hyperalgesia (21–23).

The lower incidence of agitation and respiratory/circulatory depression observed in the study group is likely attributable to the combined central and cardiovascular effects of the two agents. Remifentanil suppresses locus coeruleus norepinephrine activity, while propofol enhances GABAergic inhibition; together, these actions establish a balanced “sedation-analgesia” state that reduces emergence delirium. The significantly lower VAS and RASS scores in the study group at 12 and 24 h post-medication further support this mechanistic interpretation. Our findings are consistent with previous studies demonstrating improved emergence profiles with balanced anesthetic regimens (24–26).

The significantly lower post-extubation NE and Ang II levels in the study group underscore the combined regimen's modulatory effects on the stress axis. TBI is known to trigger HPA axis hyperactivity, resulting in catecholamine and angiotensin system dysregulation. Consistent with published reference ranges (NE: 149–564 pg/ml in resting adults; Ang II: ≤ 52 ng/L), both groups exhibited elevated stress biomarker levels, with the study group showing a more favorable profile. Remifentanil attenuates NE secretion by inhibiting nociceptive transmission and sympathetic tone, while propofol suppresses Ang II production via ACE inhibition (27–29, 33, 34).

From a neuroprotective perspective, remifentanil reduces the cerebral metabolic rate of oxygen (CMRO_2_), limiting lactate accumulation and free radical generation. Propofol has been shown to stabilize cerebral perfusion pressure (CPP) and may exert anti-inflammatory effects by modulating microglial activity and cytokine production in experimental TBI models (30). These complementary actions suggest a potential neuroprotective synergy, though direct clinical evidence remains limited.

The absence of significant differences in GCS scores at 3-month follow-up warrants cautious interpretation. Prior studies have suggested that combined anesthesia may facilitate early neurological recovery through mechanisms including ICP reduction, excitotoxicity attenuation, and hemodynamic stability maintenance (31, 32). However, the retrospective design and relatively short follow-up period in the present study preclude definitive conclusions regarding long-term neurofunctional outcomes.

### 4.1 Limitations

This retrospective single-center study has several inherent limitations. First, data completeness depended on the quality of clinical documentation, and potential confounders such as intraoperative fluid management and temperature regulation strategies could not be fully accounted for. Second, anesthesia allocation was not randomized but determined by the attending anesthesiologist in consultation with the surgical team, which may introduce selection bias. Although propensity score matching was performed as a *post-hoc* exploratory adjustment for age, GCS score, and other measured covariates, residual confounding from unmeasured factors (e.g., preoperative anxiety, substance use history) cannot be excluded.

## 5 Conclusion

The remifentanil-propofol combination significantly improves perioperative anesthesia quality in TBI patients, as evidenced by reduced agitation, attenuated stress responses, and favorable pain control. Although long-term neurological benefits remain to be established, the present findings support the clinical utility of this combined regimen, particularly in patients at high risk for perioperative complications.

## Data Availability

The original contributions presented in the study are included in the article/supplementary material, further inquiries can be directed to the corresponding authors.
